# Exploring the Volatile Fingerprinting of Young Portuguese Monovarietal Red Wines by HS-SPME-GC×GC-TOFMS: A Five-Year Study

**DOI:** 10.3390/molecules30244814

**Published:** 2025-12-18

**Authors:** Sousa Gastão-Muchecha, Nuno Martins, Raquel Garcia, Maria João Cabrita

**Affiliations:** 1 MED (Mediterranean Institute for Agriculture, Environment and Development), IIFA (Institute for Research and Advanced Training), Universidade de Évora, Pólo da Mitra, Ap. 94, 7006-554 Évora, Portugal; sousa.gastao@uevora.pt; 2 MED (Mediterranean Institute for Agriculture, Environment and Development) & CHANGE—Global Change and Sustainability Institute, Universidade de Évora, Pólo da Mitra, Ap. 94, 7006-554 Évora, Portugal; nmartins@uevora.pt (N.M.); raquelg@uevora.pt (R.G.); 3 Departamento de Fitotecnia, Escola de Ciências e Tecnologia, Universidade de Évora, Pólo da Mitra, Ap. 94, 7006-554 Évora, Portugal

**Keywords:** volatile organic compounds, terpenes, SPME, multivariate analysis, Alentejo wines, varietal typicity

## Abstract

The aroma of wine is a defining quality attribute, determined mainly by volatile organic compounds (VOCs) originating from grape metabolism, fermentation, and maturation. This study aimed to characterize the VOC composition of young monovarietal red wines from the Alentejo region (Portugal), produced from Alicante Bouschet, Touriga Nacional, and Trincadeira across five consecutive vintages (2020–2024). Headspace solid-phase microextraction (HS-SPME) coupled with comprehensive two-dimensional gas chromatography–time-of-flight mass spectrometry (GC×GC-ToFMS) was applied for VOC profiling, followed by multivariate statistical analyses. A strict identification and reproducibility criterion was applied to ensure longitudinal consistency over the five vintages. MANOVA analysis revealed significant effects (*p* < 0.001) of both grape variety and vintage on VOC distribution. Esters were the most abundant and discriminant group, while aldehydes and terpenes contributed markedly to varietal differentiation. Alicante Bouschet wines were associated with fruity ethyl esters, Touriga Nacional with monoterpenes (citronellol, terpinolene, α-farnesene) and aromatic alcohols, and Trincadeira with aldehydes and sesquiterpenes. Canonical discriminant analysis (CDA) achieved clear separation among varieties and vintages, with the first two canonical functions accounting for over 70% of the total variance. Heatmap analysis highlighted distinctive terpene and C13-norisoprenoid profiles across samples. These findings demonstrate the pivotal role of VOCs in defining Alentejo wine typicity and support their use as chemical markers for authenticity and PDO valorization.

## 1. Introduction

The aroma of wine is one of the most important quality factors, as it directly influences how consumers perceive and prefer it. This aroma originates from a complex blend of volatile organic compounds (VOCs) produced during grape metabolism, yeast fermentation, and subsequent aging processes [1]. More than 800 VOCs have been identified in wines so far, including esters, higher alcohols, volatile fatty acids, terpenes, C13-norisoprenoids, and carbonyl compounds, among others. Each of these chemical groups contributes to the overall aroma of wine in varying amounts, shaping its sensory complexity and influencing varietal and vintage distinctions [2,3].

The composition of VOCs is strongly influenced by viticultural factors such as grape variety, terroir, climatic conditions, and vineyard practices, as well as enological decisions, including fermentation, yeast strain, and post-fermentative treatments [1,4]. In this context, monovarietal wines provide a unique framework for studying the relationship between grape genetics and volatile composition, expressing the chemical characteristics of the grape variety. Portuguese red grape varieties are particularly relevant due to their oenological potential, contribution to the identity of Iberian wines, and growing interest in international markets [5].

The accurate characterization of VOC profiles requires analytical methodologies that are both sensitive and selective, capable of capturing the chemical diversity of wine matrices. Since the late 1990s, headspace solid-phase microextraction (HS-SPME) coupled with gas chromatography-mass spectrometry (GC–MS) has become a widely used method for analyzing wine volatiles [6,7]. However, HS-SPME results are highly dependent on extraction parameters, including fiber coating, extraction time, temperature, and salt addition, highlighting the need for rigorous method optimization and standardization to ensure reproducibility and comparability [3]. More recently, comprehensive two-dimensional gas chromatography combined with time-of-flight mass spectrometry (GC×GC-ToFMS) has emerged as a powerful approach for profiling VOCs at trace levels, resolving coelution effects that could occur on one-dimensional separations, and enabling the detection of minor compounds relevant to varietal differentiation [5,8].

Previous studies have addressed the volatile composition of monovarietal wines from Alentejo [9] over two consecutive harvests. Other studies focused on monovarietal wines from the Bairrada and Douro regions in specific technological/intervention contexts (e.g., effects of malolactic fermentation on Trincadeira wines; kaolin spraying on Touriga Nacional vines) [10,11,12,13], among others. However, due to advances in analytical techniques, a systematic study of the volatile composition of Portuguese monovarietal wines from Alentejo, particularly of young wines, can be revisited, as information remains scarce. Filling this gap is essential not only to refine the chemical understanding of the typicality of Portuguese wine in the context of the Alentejo but also to support claims of authenticity, quality control, and the promotion of Protected Designation of Origin (PDO) products in international markets.

In this context, the present study aims to characterize the volatile organic compound (VOC) profiles of young monovarietal red wines from the Alentejo region of Portugal, produced from Alicante Bouschet, Touriga Nacional, and Trincadeira grapes, over five consecutive years. HS-SPME coupled with GC×GC-ToFMS was applied to identify varietal markers and evaluate the differentiating potential of these cultivars within the Alentejo viticultural context.

## 2. Results and Discussion

### 2.1. Overview by Chemical Classes (HS-SPME-GC×GC-ToFMS)

The VOC profiles of young monovarietal red wines from the Alentejo region, analyzed over five consecutive vintages (2020–2024) using HS-SPME-GC×GC-ToFMS, revealed significant differences among cultivars (Alicante Bouschet, Touriga Nacional, and Trincadeira), and between vintages. The results of the MANOVA analysis (Table 1) confirm that both varietal and year factors strongly influenced the distribution of volatile constituents, with several compounds showing significant effects for Year, Variety, and the Year × Variety interaction. Mean and standard deviation values are in the Supplementary Materials (Table S1). Esters constituted the most abundant and discriminant group of volatiles. Compounds such as acetic acid, 2-methylpropyl ester (1), butanoic acid, ethyl ester (2), and propanoic acid, 2-hydroxy-, ethyl ester (3) all displayed significant effects of Year, Variety, and their interaction (*p* ≤ 0.01). These esters are known to contribute fruity and floral nuances, particularly in young wines [10]. Other esters, such as octanoic acid ethyl ester (19), benzeneacetic acid ethyl ester (20), and isopentyl hexanoate (21), also showed strong varietal differentiation. Their significance across vintages highlights the modulation of ester biosynthesis by climatic and fermentation conditions typical of the Alentejo region [11]. Among the alcohols, 1-hexanol (47) and 1-heptanol (50) were highly significant across all factors (*p* ≤ 0.001), confirming their importance as varietal markers. Notably, linalool (53) and phenylethyl alcohol (54) were strongly associated with Touriga Nacional, contributing to its well-documented floral and aromatic typicity [12].

Trincadeira was distinguished by higher levels of aldehydes, particularly benzaldehyde (57), benzeneacetaldehyde (58), and Nonanal (59), which exhibited significant varietal and Year × Variety effects. These compounds contribute almond-like and nutty nuances, consistent with the more complex sensory profile of this cultivar [13]. The varietal distinction of Touriga Nacional was reinforced by terpenic compounds such as γ-terpinene (67), terpinolene (68), and α-farnesene (71), which were significant across year and variety effects. These volatiles are associated with floral and herbal sensory notes, aligning with Touriga Nacional established aromatic profile [14]. Although norisoprenoids were less abundant, naphthalene derivatives (77) also showed significant varietal discrimination.

The results indicate that specific volatile markers robustly explain varietal differentiation in young Alentejo wines (2020–2024). Alicante Bouschet was primarily associated with higher levels of fruity ethyl esters and acetate derivatives, which align with its fresh, fruit-forward aromatic profile. This is consistent with previous findings that highlight esters as key contributors to the sensory profile of this cultivar [10,12]. Touriga Nacional was distinguished by elevated levels of monoterpenes (e.g., citronellol, α-farnesene, terpinolene, 3-carene) and aromatic alcohols, such as phenylethyl alcohol, which reinforces its floral and perfumed character, widely reported in the literature [15,16,17]. In contrast, Trincadeira showed higher contributions from odor-active aldehydes (e.g., benzaldehyde and nonanal), which impart a more complex, spicy, and structured aromatic profile, consistent with previous studies on this variety [18]. Overall, these compound classes emerged as key discriminants among the three cultivars, highlighting the pivotal role of the volatile fraction in varietal characterization within the Alentejo viticultural context.

Overall, the results shown in Table 1 confirm that each cultivar has a specific set of volatile markers: esters (compounds 1 to 23) for Alicante Bouschet, terpenes and aromatic alcohols (compounds 48, 54, to 56) for Touriga Nacional, and aldehydes (compounds 57, 58, and 59) for Trincadeira. These findings align with previous reports on the chemical basis of varietal typicity but extend the knowledge to young Alentejo wines across multiple consecutive vintages [19,20]. The significant Year effect underscores the influence of climate and viticultural conditions on aroma profiles, emphasizing the importance of vintage characterization for PDO valorization. It should be noted that the 77 VOCs reported correspond to a subset of analytically robust compounds detected consistently across the five harvests and that meet strict identification criteria (mass spectral quality, reproducible triplicate detection, and reliable retention index behavior). Although GC×GC-ToFMS created a larger number of deconvoluted raw features, only those reliably identified VOCs were retained for annotation. From this identified subset, only compounds that showed statistically significant effects in MANOVA were used in the multivariate modeling of Variety Effect and Harvest Effect (CDA), as specified in the Section 3.5. In contrast, the heat maps presented in Section 2.3 include all terpenes and all identified C13-norisoprenoids, regardless of their statistical significance, since their purpose was to illustrate general distribution patterns and trends in relative abundance between varieties and harvests, rather than to perform inferential tests.

### 2.2. Canonical Discriminant Analysis (CDA)

The canonical discriminant analysis (CDA) was performed to evaluate the ability of volatile organic compounds (VOCs) to discriminate between samples based on vintage year and variety. This multivariate approach is widely used in wine research because it simultaneously integrates the variability of multiple volatile classes, creating discriminant functions that maximize separation between predefined groups [10,21,22]. Each discriminant function was evaluated for its statistical significance in terms of Wilks’ lambda factor. The range of Wilks’ lambda is from 1 (zero discrimination power) to 0 (perfect discrimination power).

#### 2.2.1. Effect of Vintage Year

**Figure 1:**
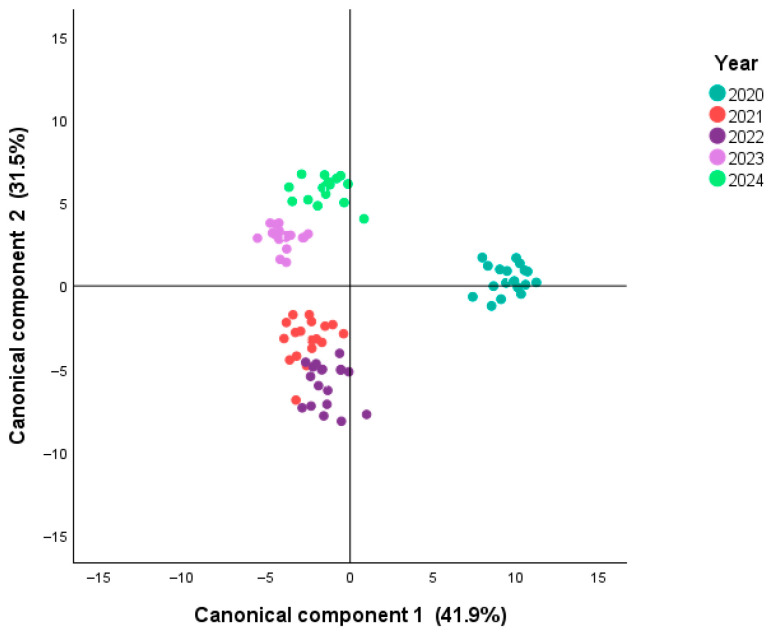
Canonical discriminant analysis (CDA) scatterplot based on the first two canonical functions (41.9% and 31.5% of total variance, respectively), showing the separation of young monovarietal red wines from Alentejo (2020–2024) according to harvest year.

A canonical discriminant analysis (CDA) scatterplot based on the first two canonical functions is depicted in Figure 1. The first two functions explain 73.4% of the total variance (41.9% and 31.5%, respectively), with canonical correlations of 0.981 and 0.974, respectively. The first two functions presented a significant Wilk’s lambda value (0.000 and 0.001 with *p* < 0.001), confirming that vintage has a strong influence on the volatile composition of young Alentejo wines. It is important to note that all wines were stored frozen at −32 °C immediately after malolactic fermentation and analyzed in a single batch. Therefore, the differences observed among vintages reflect biochemical and climatic influences rather than post-fermentative aging effects such as ester hydrolysis or terpene oxidation. Figures S2–S6 (Supplementary Materials) show the monthly temperature (average, minimum and maximum) and precipitation for each year. The differences observed for each year can also help to understand the CDA results.

These findings align with previous reports showing that vintage conditions (e.g., temperature, rainfall, and sunlight) significantly affect grape ripening, yeast metabolism, and, consequently, the formation of esters, alcohols, and terpenes [12,23]. For instance, [24] observed that Montepulciano wines displayed distinct volatile profiles depending on the harvest year, with esters being particularly sensitive to climatic variability. Likewise, [25] demonstrated that the volatile composition, combined with mineral elements and metabolites, provided strong discriminatory power for classifying Chinese wines by geographical origin, underscoring the robustness of multivariate chemometric approaches for authenticity validation. These results, supported by eigenvalue and Wilks’ Lambda statistics, confirm the robustness of vintage-related discrimination in the volatile composition of Alentejo wines. Globally, these results reinforce that VOCs are not only responsible for varietal signatures but also provide a reliable fingerprint of climatic and enological variability across consecutive vintages.

#### 2.2.2. Effect of Variety

**Figure 2:**
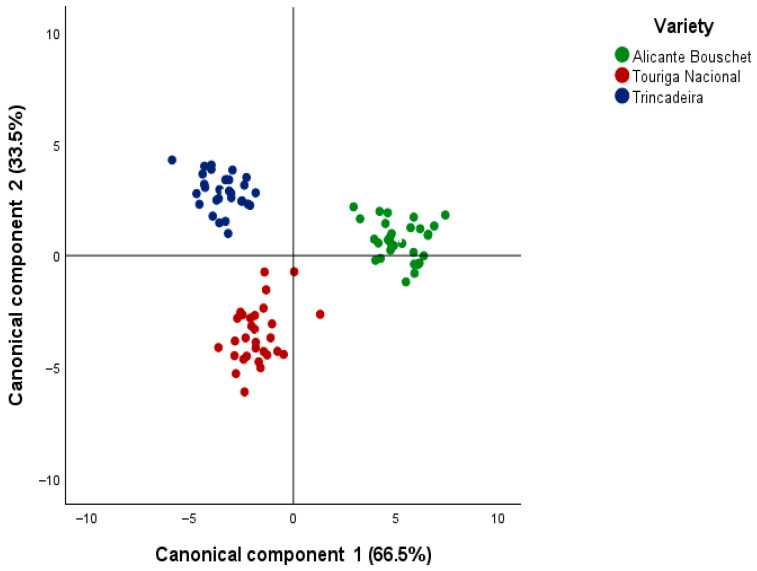
Canonical discriminant analysis (CDA) scatterplot based on the first two canonical functions (Function 1: 66.5%; Function 2: 33.5%), illustrating the clear separation of young monovarietal red wines from Alentejo (2020–2024) according to grape variety (Alicante Bouschet, Touriga Nacional, and Trincadeira) as determined from their VOC profiles.

When CDA was applied to discriminate wines according to grape variety, two canonical functions were obtained, explaining 100% of the total variance, with the first function explaining 66.5% and the second one explaining 33.5% of the total variance among varieties. Canonical correlations were again high (0.967 and 0.938), with Wilks’ lambda values of 0.08 and 0.120 (*p* < 0.001) confirming the model’s strong discriminative power. The scatterplot of functions 1 and 2 (Figure 2) revealed distinct clustering of Alicante Bouschet, Touriga Nacional, and Trincadeira, with no overlap between groups, in spite of some differences found among wines’ oeological parameters (Table S2 Supplementary file).

This clear varietal separation underscores the robustness of VOC markers in distinguishing Portuguese grape varieties. Similar observations have been reported in Portuguese wines, where CDA successfully differentiated varietal wines based on esters, higher alcohols, and terpenes [16]. More specifically, Touriga Nacional has been repeatedly associated with elevated levels of monoterpenes, contributing to its floral and aromatic typicity [17]. In contrast, Trincadeira wines are distinguished by odor-active aldehydes and volatile phenols, reflecting their more complex aromatic profile [18]. In contrast, Trincadeira wines are distinguished by odor-active aldehydes and volatile phenols, reflecting their more complex aromatic profile [18]. In a broader perspective, the application of chemometric tools to Portuguese monovarietal wines has been shown to enhance the interpretation of VOC data, as illustrated by [12], who employed aroma networks to differentiate varietal profiles. At the methodological level, GC×GC-ToFMS has been widely validated in the literature for discriminating wines by grape variety based on volatile data, reinforcing the robustness of the present results [10].

### 2.3. Terpenes and C13-Norisoprenoids as Varietal and Vintage Differentiators

**Figure 3:**
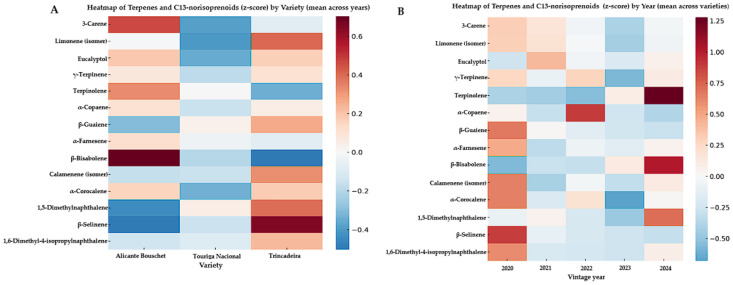
Heatmaps of terpenes and C_13_-norisoprenoids (z-score) in young monovarietal Alentejo red wines analyzed by HS-SPME-GC×GC-ToFMS (2020–2024). (**A**) Compound profiles averaged across vintages, showing differences by grape variety: Alicante Bouschet, Touriga Nacional, and Trincadeira. (**B**) Compound profiles averaged across varieties, highlighting variation across vintages (2020–2024). Warmer colors indicate higher relative abundance (positive z-scores), while cooler colors represent lower abundance (negative z-scores).

The heatmap analysis of the terpene and C13-norisoprenoid dataset (Figure 3) revealed clear differentiation patterns both by grape variety and by vintage year, reaffirming the significance of both terpenoids and norisoprenoids as complementary markers of aromatic typicity in young Alentejo wines. Regarding the varietal effect (Figure 3A), Touriga Nacional was characterized by higher relative abundances of monoterpenes such as 3-carene, terpinolene, γ-terpinene, and α-farnesene, compounds strongly associated with floral and aromatic notes, consistent with its well-recognized typicity [15,17]. Trincadeira, in contrast, exhibited sesquiterpenes, including α-copaene, β-guaiene, and β-bisabolene, which are associated with earthy, spicy, and resinous aromas, contributing to its more complex and structured aromatic profile [18]. Meanwhile, Alicante Bouschet, although less abundant in floral monoterpenes, displayed significant levels of d-limonene and eucalyptol, reinforcing its citrus freshness and herbal nuances, consistent with its fruit-driven typicity. In addition, some C13-norisoprenoids identified in this study, namely Naphthalene, 1,5-dimethyl-, β-Selinene, and Naphthalene, 4-isopropyl-1,6-dimethyl, also displayed varietal clustering, suggesting cultivar-dependent differences in carotenoid degradation and secondary metabolite pathways. Although their direct sensory impact is less well studied than that of well-known norisoprenoids such as β-damascenone or β-ionone, their occurrence reinforces the relevance of norisoprenoid metabolism as a contributing factor to varietal typicity and vintage modulation in wines [26,27].

Focusing on vintage variation (Figure 3B), the heatmaps revealed marked fluctuations in terpene abundance across the 2020–2024 harvests, highlighting the strong influence of climatic variability and environmental conditions on terpene biosynthesis [28,29]. Certain monoterpenes (e.g., γ-terpinene) were more prominent in warmer vintages, whereas sesquiterpenes (e.g., β-bisabolene) and certain norisoprenoids showed higher relative abundance in cooler years, suggesting dynamic adaptation of secondary metabolite pathways under terroir conditions [30]. This aligns with studies showing that C13-norisoprenoid levels in grapes respond to sunlight exposure and canopy management, due to their origin from carotenoid breakdown [27,31].

These findings reinforce current knowledge in wine flavor chemistry, where monoterpenes (e.g., linalool, geraniol, nerol, citronellol, α-terpineol) are widely recognized as major contributors to floral and citrus notes typical of aromatic cultivars, while sesquiterpenes contribute earthy, woody, and spicy nuances, adding aromatic depth and typicity [28,29]. C13-norisoprenoids, such as β-damascenone and β-ionone, thanks to their extremely low sensory thresholds, exert a significant impact on aroma even at trace levels [32,33]. While these visual patterns provided strong varietal and vintage clustering, ANOVA confirmed that only a subset of these compounds reached statistical significance. Overall, the combined varietal and vintage effects observed in Alentejo wines underscore the dual role of genetic background and environmental modulation in shaping the volatile profile, supporting their relevance as enological markers and as tools for the valorization of PDO wines from the region [34,35].

## 3. Materials and Methods

### 3.1. Wine Samples

Wines studied in this work were produced in the University of Évora winery from Alicante Bouschet, Touriga Nacional, and Trincadeira grapes harvested from the Évora University vineyard, ensuring that vines were grown under identical soil, climate, and cultivation conditions across five consecutive vintages (2020–2024). Grapes were processed at Évora University Experimental Winery, using the same vinification protocol for all varieties. Briefly, grapes were destemmed, crushed, and vinified independently in 50 L stain steel deposits. Only SO_2_ was added (30 mg L^−1^) at the beginning of alcoholic fermentation, using a commercial 6% aqueous solution of sodium bisulfite (SAI, SOLFOX 6 Nº CE 231-870-1). The fermenting musts were punched down twice a day to promote pomace contact, and when the amount of reduced sugar was less than 3 gL^−1^, free-run wine was separated from pomace without pressing. Malolactic fermentation (MLF) took place in 25 L glass vessels. Immediately after the completion of malolactic fermentation, all wines from each vintage were frozen at −32 °C to prevent post-fermentative chemical evolution and ensure comparability across vintages. All wines were produced in duplicate. In addition, meteorological data (monthly average, maximum and minimum temperatures, and precipitation) for the 2020–2024 harvests were obtained from the Mitra weather station (ICTERRA Meteorological Data Portal; Évora, Portugal). Annual climate summaries for each harvest are presented in Supplementary Figures S2–S6.

### 3.2. Oenological Parameters

Grape classical parameters such as °Brix, pH, and titratable acidity (TA, g/L of tartaric acid), along with the classical parameters of wines, such as alcoholic degree (°A), pH, titratable acidity (TA, g/L of tartaric acid), and volatile acidity (VA, g/L of acetic acid), were analyzed by OIV methods [36]. For each vintage and grape variety, the oenological parameters were measured from the two separate vinification replicates. The complete set of average oenological data, including alcohol content, reducing sugars, titratable and volatile acidity, pH, and free/total SO_2_, is provided in Supplementary Table S2.

### 3.3. HS-SPME Methodology

A carboxen/divinylbenzene/polydimethylsiloxane fiber (CAR/DVB/PDMS), 1 cm, 50/30 μm film thickness, supplied from Supelco (Bellefonte, PA, USA), was used for HS-SPME extractions. Fiber blanks were run periodically; that is, a blank was carried out before the injection of the first sample of grapes, and the remaining blanks were carried out every three injections to ensure the absence of contaminants and/or carryover. HS-SPME extraction was adapted from a previously developed procedure [5] with some modifications. In a 20.0 mL SPME flask sealed with a Teflon-coated rubber septum/magnetic screw cap, 5 mL of wine was weighed, then 0.6 g of sodium chloride (NaCl) was added. Additionally, as noted in the literature, NaCl increases the ionic strength of the samples, thereby reducing the solubility of the compounds and altering their partition coefficients, thereby enhancing analyte extraction. The vial was equilibrated for 5 min at 40 °C and then extracted for 30 min at the same temperature. The thermal desorption of the analytes was performed by exposing the fiber in the GC injection port at 260 °C for 3 min in split mode with a split ratio of 10:1. All measurements were performed in triplicate. All samples from the five vintages were thawed and analyzed within a single analytical batch using identical instrument conditions, the same column, and the same detector settings. This approach eliminates inter-batch variability caused by instrument drift, column aging, or changes in sensitivity. This extraction and detection workflow prioritized chromatographic and spectral reproducibility to ensure that only robustly detected VOCs were forwarded for identification and statistical evaluation.

### 3.4. GC×GC-TOFMS Analysis

The analyses were performed on a GC×GC-ToFMS system consisting of an Agilent 8890 GC System (Shanghai, China) with a BenchTOF-Select detector (Markes International, Bridgend, UK). An automatic sampler injector (CTC Analysis autosampler, PAL-System, SepSolve Analytical, Zwingen, Switzerland) was used, and the data were acquired and analyzed using ChromSpace (Markes International, Bridgend, UK). Chromatographic separation was achieved with INSIGHT™ flow modulator (SepSolve Analytical, Waterloo, ON, Canada), equipped with a loop with 50 μL, a BPX5 column (20 m length × 0.18 mm i.d. and 0.18 μm film thickness, from SGE GC column, Trajan, Australia) as first-dimension (^1^D) and a BPX50 column (5 m length × 0.25 mm i.d. and 0.1 μm film thickness, from SepSolve Analytical, Waterloo, Canada) as second-dimension (^2^D).

The modulation period (P_M_) used was 5 s, and the flush time (FT) was 200 ms. The oven temperature program began at 40 °C, held for 3 min, then raised at 3 °C/min to 150 °C, followed by a 4 °C/min increase to 200 °C, and then a 10 °C/min increase to 260 °C, with a hold time of 5 min. Helium was used as the carrier gas, with a flow rate of 0.5 mL/min in the first column and 20 mL/min in the second column. The MS transfer line and source temperatures were set to 270 °C. To determine the characteristic mass fragments, electron ionization (EI) mass spectra of the analytes were recorded at 70 eV in full scan mode, from 30 to 400 *m*/*z*, with a data acquisition frequency of 50 Hz. The linear retention index values were calculated by analyzing a commercial hydrocarbon mixture (C8–C20, Supelco, Bellefonte, PA, USA) under the same chromatographic conditions. A mixture of terpenes called MegaMix #1 (Restek, Bellefonte, PA, USA) was injected to help identify terpenes. The volatile compounds were first identified by matching mass spectra with those of reference compounds in the NIST mass spectral library (NIST MS Search Program Version 2020). Additionally, structural and molecular weight considerations were taken into account, and the calculated LRIs were compared with those reported in the literature. The relative abundance of each compound was calculated as a percentage of its respective peak area relative to the total chromatographic peak area (% A). Although the raw GC×GC-ToFMS chromatograms contained several hundred deconvoluted peaks per sample, only volatile compounds showing reproducible detection in triplicate injections, high-quality mass-spectral matching (NIST), consistent retention-index behavior, and occurrence across all five harvests were retained for annotation. This conservative selection ensured longitudinal comparability and prevented the inclusion of low-abundance or poorly resolved features that could compromise the robustness of the multivariate analyses. A representative GC×GC contour plot illustrating peak distribution and chromatographic separation under these conditions is provided in Supplementary Figure S1.

### 3.5. Statistical Analysis

The statistical analyses were performed using NCSS 11 Statistical Software, version 11.0.24 (2020) (LLC., Kaysville, UT, USA). A multivariate analysis of variance (MANOVA) was used to evaluate the significance of the factors “variety,” “year,” and their interaction (“variety × year”) on the volatile composition of the wines. Canonical discriminant analysis (CDA) was subsequently conducted using IBM SPSS Statistics version 30 (IBM Corp., Armonk, NY, USA). Only the variables that showed significant differences in the MANOVA analysis were used. Eigenvalues and Wilks’ λ were employed to determine the discriminatory power and statistical significance of the canonical functions. Complementarily, data visualization was carried out in Google Colab, adapted for execution in a Jupyter environment using specialized Python 3.10 and Matplotlib 3.10 libraries. Heatmaps based on z-score-normalized values were constructed to illustrate the relative abundance of terpene and C13-Norisoprenoid compounds across varieties and vintages (2020–2024). This combined statistical and computational approach enabled both robust hypothesis testing and intuitive graphical interpretation of the effects of varietal and vintage on the volatile fraction of young Alentejo wines.

## 4. Conclusions

This study showed that volatile organic compound (VOC) profiles are reliable markers for distinguishing young monovarietal red wines from Alentejo across five vintages (2020–2024). Both grape variety and vintage year significantly affected the volatile composition, emphasizing the dual influence of genetic and environmental factors. Among the classes of volatiles, esters, terpenes, and aldehydes stood out as key contributors to varietal identity, with terpenes and sesquiterpenes particularly relevant in differentiating Touriga Nacional, Trincadeira, and Alicante Bouschet. Complementary heatmaps revealed clear clustering patterns for terpenes and C13-norisoprenoids, providing additional discriminatory power across varieties and vintages.

The findings highlight the importance of VOCs as chemical fingerprints that support varietal authenticity and provide a strong basis for Protected Designation of Origin (PDO) valorization strategies in Alentejo wines. Additionally, the demonstrated impact of vintage underlines the sensitivity of volatile profiles to climate conditions, emphasizing the role of terroir in shaping wine aroma.

Future research should combine sensory analysis with non-volatile compounds to strengthen the link between chemical markers and perceived wine quality, thereby enhancing the potential of VOC profiling as a comprehensive tool for authenticity assessment.

## Figures and Tables

**Table 1:** The results MANOVA for the volatile compounds found in Alicante Bouschet, Touriga Nacional, and Trincadeira wines across five consecutive vintages (2020 to 2024).

	Compound Name	^1^t_R_ (min) ^a^	^2^t_R_ (secs) ^b^	LRI _lit_ ^c^	LRI _calc_ ^d^	Ions *m*/*z* ^e^	MANOVA		
Compound No.	Esters						Y	V	Y × V
1	Acetic acid, 2-methylpropyl ester	10.0537	2.9067	755	-	43/56/73	*	*	*
2	Butanoic acid, ethyl ester	11.0833	2.9684	784	-	71/43/88	***	***	***
3	Propanoic acid, 2-hydroxy-, ethyl ester	11.7749	3.2479	799	-	45/75	**	**	***
4	Butanoic acid, 2-methyl-, ethyl ester	13.4325	2.9758	837	-	57/102/85	***	***	***
5	Butanoic acid, 3-methyl-, ethyl ester	13.638	3.0022	838	-	88/85/57	***	***	***
6	Hexanoic acid, methyl ester	17.3661	3.1	907	-	74/87/99	***	***	**
7	3-Hexenoic acid, ethyl ester	20.7199	3.2081	986	993	68/88/41	n.s.	n.s.	**
8	Hexanoic acid, ethyl ester	21.2749	3.043	999	1001	88/60/41	***	n.s.	**
9	Acetic acid, hexyl ester	22.0117	3.0209	1000	1016	43/56	***	***	***
10	2-Hexenoic acid, ethyl ester	22.6897	3.012	1019	1030	88/115/55	n.s.	***	n.s.
11	2-Hexenoic acid, ethyl ester, (isomer)	23.8555	2.7693	1038	1053	97/99/55/73	*	n.s.	n.s.
12	Butanoic acid, pentyl ester	24.3037	2.9986	1078	1062	71/43/55	**	n.s.	*
13	Hexanoic acid, propyl ester	26.25	3.0241	1080	1100	43/99/117	***	***	***
14	Acetic acid, heptyl ester	27.1871	3.0121	1094	1119	43/56/70	***	***	***
15	Octanoic acid, methyl ester	27.8138	3.0376	1107	1131	74/87/55/43	***	***	***
16	Hexanoic acid, 2-methylpropyl ester	29.1082	2.9377	1138	1156	99/56/117/71	*	n.s.	***
17	Butanedioic acid, diethyl ester	30.7758	3.6011	1191	1189	101/129/45	***	**	**
18	7-Octenoic acid, ethyl ester	30.9981	3.0956	1187	1193	88/55	***	*	***
19	Octanoic acid, ethyl ester	31.4556	2.9959	1193	1203	88/101/57/41	**	**	***
20	Benzeneacetic acid, ethyl ester	33.7091	3.0513	1218	1250	91/164/65/43	**	**	***
21	2-Hexenoic acid, ethyl ester, (isomer)	33.9751	2.9463	1234	1256	70/43/55/99	***	**	*
22	Acetic acid, 2-phenylethyl ester	34.6467	3.8479	1250	1270	104/91/43	***	**	***
23	2-Propenoic acid. octyl ester	35.3333	2.9842	1273	1284	56/70/43	*	n.s.	**
24	Octanoic acid, propyl ester	35.9167	2.968	1277	1297	145/127/61/43/73	n.s.	n.s.	n.s.
25	Nonanoic acid, ethyl ester	36.75	2.9274	1279	1315	88/101/141/73	***	***	***
26	Decanoic acid, methyl ester	37.3986	2.9831	1308	1329	74/87/143	n.s.	n.s.	n.s.
27	Caprylic acid isobutyl ester	38.395	2.8964	1334	1352	57/127/41	***	***	**
28	Decanoic acid, ethyl ester	38.8993	2.9118	1379	1363	88/101/73/55	n.s.	n.s.	n.s.
29	Decanoic acid, ethyl ester	40.5641	2.918	1381	1400	88/101/43/55	***	n.s.	**
30	Butanedioic acid, ethyl 3-methylbutyl ester	42.1006	3.2639	1422	1439	101/129/55/43	***	n.s.	n.s.
31	Octanoic acid, 3-methylbutyl ester	42.6818	2.8371	1450	1453	70/127/55	***	***	***
32	Undecanoic acid, ethyl ester	43.119	2.8092	1479	1464	88/101/55/43	n.s.	n.s.	n.s.
33	n-Capric acid isobutyl ester	46.2815	2.709	1531	1550	56/155/173	**	n.s.	n.s.
34	Dodecanoic acid, ethyl ester	46.6511	2.6258	1578	1560	88/101/83/55	n.s.	n.s.	n.s.
35	Octanoic acid, hexyl ester	47.5833	2.7529	1579	1587	145/85/43/127	***	**	***
36	Pentadecanoic acid, 3-methylbutyl ester	49.6095	2.6785	1630	1651	70/43/155	***	***	***
37	Tridecanoic acid ethyl ester	50.1809	2.6639	1677	1669	88/101/55/43	n.s.	n.s.	*
38	Tetradecanoic acid, ethyl ester	53.8074	2.4483	1778	1797	88/101/73/55/43	n.s.	*	**
39	Tetradecanoic acid, isopropyl ester	54.4262	2.3287	1813	1826	43/60/102/129	n.s.	*	**
40	Dodecanoic acid, 3-methylbutyl ester	54.9399	2.3473	1829	1852	70/55/43/60/85	**	n.s.	***
41	Pentadecanoic acid, ethyl ester	55.4842	2.943	1879	1879	88/101/43	n.s.	*	***
42	9-Hexadecenoic acid, ethyl ester	57.2039	2.2213	1955	1982	55/69/88/41	n.s.	n.s.	n.s.
43	Hexadecanoic acid, ethyl ester	57.4167	2.1617	1979	1995	88/101/43/115	***	**	***
44	Linoleic acid ethyl ester	59.5966	2.943	2141	-	67/55/81/41	*	**	*
45	Octadecanoic acid, ethyl ester	60.1667	2.2752	2180	-	88/101/43/55	n.s.	n.s.	n.s.
	**Alcohols**								
46	1-pentanol	10.758	2.7464	756	-	55/43/70	n.s.	n.s.	n.s.
47	1-Hexanol	14.6197	3.1059	854	-	56/69/43	**	***	***
48	1-Butanol, 3-methyl-, acetate	14.7989	3.0016	859	-	43/55/70	**	n.s.	***
49	1-Butanol, 3-methyl-, propanoate	19.6807	2.9914	952	979	57/70/43	n.s.	n.s.	n.s.
50	1-Heptanol	19.9474	3.0821	953	982	70/56/41	***	***	***
51	1-Propanol, 3-(methylthio)	20.8656	3.9684	982	995	106/61/47	***	***	***
52	1-Octanol	25.2056	3.0561	1057	1080	56/41/70/85	***	n.s.	***
53	Linalool-I	26.6667	3.0547	1086	1109	93/55/41	***	***	***
54	Phenylethyl Alcohol	27.8923	4.002	1110	1133	91/122/77	***	***	***
55	Citronellol	33.0	3.1438	1211	1235	69/67/55/41/81	***	*	***
56	1-Decanol	35.3333	3.783	1274	1284	70/55/43/83/97	n.s.	n.s.	n.s.
	**Aldehydes**								
57	Benzaldehyde	20.0358	4.002	961	984	106/77/51	***	***	***
58	Benzeneacetaldehyde	24.3638	4.0795	1050	1063	91/120/65	n.s.	***	***
59	Nonanal	27.0	3.0509	1090	1115	57/41/70/82	***	**	**
	**Carboxylic acids**								
60	Heptanoic acid	20.4092	3.037	982	989	60/73/41	**	n.s.	***
61	Oleic Acid	59.8254	2.3165	2116	-	55/43/69/83	**	***	n.s.
	**Ketones**								
62	Butyrolactone	17.6889	5.3841	908	-	42/86/85	***	***	***
63	Cyclohexanone, 2,2,6-trimethyl-	34.6467	3.8479	1036	1046	82/69/56	***	***	***
	**Terpenes**								
64	3-Carene-I	22.6337	4.4006	1006	1029	93/91/121/136	***	***	***
65	Limonene-I	22.9578	2.8899	1020	1035	68/93/136	n.s.	n.s.	n.s.
66	Eucalyptol	23.25	3.04781	1022	1041	43/71/91/93/139	n.s.	***	***
67	γ-Terpinene-I	24.5	2.990	1050	1066	93/77/121/136	**	n.s.	***
68	Terpinolene-I	5.9494	3.0399	1079	1094	121/93/136/79	**	**	**
69	α-Copaene	39.75	2.957	1380	1382	105/119/93/161	*	n.s.	**
70	β-Guaiene	44.5833	2.9528	1494	1501	161/105/119	n.s.	n.s.	**
71	α-Farnesene	44.8955	2.8158	1496	1510	93/55/79/69/107/119	*	***	***
72	β-Bisabolene	45.1488	2.8428	1500	1517	69/93/41	n.s.	n.s.	**
73	Calamenene (isomer)	45.9424	3.0622	1512	1540	159/129/105	*	n.s.	**
74	α-Corocalene	49.2314	3.2469	1607	1638	185/157/143/128	n.s.	n.s.	n.s.
	**C_13_-Norisoprenoids**								
75	1,5-Dimethylnaphthalene	42.5833	3.703	1439	1451	156/141/70	n.s.	n.s.	n.s.
76	β-Selinene	44.0721	3.6475	1482	1488	93/79/55/67/107	n.s.	n.s.	n.s.
77	1,6-Dimethyl-4-isopropylnaphthalene	51.1347	3.3451	1682	1700	181/198	***	**	***

Y: Year; V: Variety; ^1^t_R_ (min) ^a^: first dimension retention time; ^2^t_R_ (secs) ^b^: second dimension retention time; LRI _lit_ ^c^: The linear retention index values from the literature for a 5% phenyl polysilphenylene-siloxane column; LRI _calc_ ^d^: The linear retention index values were calculated through analysis of the commercial hydrocarbon mixture (C8–C20); ions *m*/*z* ^e^: indicates the characteristic fragment ions used for compound identification by mass spectrometry; I: identification confirmed by reference standard (retention index and mass spectrometry); Statistically significant at * *p* ≤ 0.05, ** *p* ≤ 0.01, and *** *p* ≤ 0.001, n.s.: Not significant, respectively.

## Data Availability

The data presented in this study are available on request from the corresponding author.

## Supplementary Materials

The following supporting information can be downloaded at: https://www.mdpi.com/article/10.3390/molecules30244814/s1, Table S1: Mean and standard deviation values; Table S2: oenological parameters; Figure S1: Representative GC×GC-ToFMS contour plots obtained for the 2020 wines of the three grape varieties studied: Alicante Bouschet (AB), Trincadeira (Trinc), and Touriga Nacional (TN); Figure S2: Monthly temperature (average, maximum, and minimum) and precipitation recorded at the Mitra weather station (Évora, Portugal) during the 2020 year; Figure S3: Monthly temperature (average, maximum, and minimum) and precipitation recorded at the Mitra weather station (Évora, Portugal) during 2021; Figure S4: Monthly temperature (average, maximum, and minimum) and precipitation recorded at the Mitra weather station (Évora, Portugal) during 2022; Figure S5: Monthly temperature (average, maximum, and minimum) and precipitation recorded at the Mitra weather station (Évora, Portugal) during 2023; Figure S6: Monthly temperature (average, maximum, and minimum) and precipitation recorded at the Mitra weather station (Évora, Portugal) during 2024.

## References

[1] Styger G., Prior B., Bauer F.F. (2011). Wine Flavor and Aroma. J. Ind. Microbiol. Biotechnol..

[2] Luo J., Zhang P., Loo Y.T., Ma J., Wu S., Marriott P.J., Howell K. (2022). Can Wine Quality Be Predicted by Small Volatile Compounds? A Study Based on Performance of Wine Show Entries and Their Volatile Profiles. Flavour Fragr. J..

[3] Tufariello M., Pati S., Palombi L., Grieco F., Losito I. (2022). Use of Multivariate Statistics in the Processing of Data on Wine Volatile Compounds Obtained by HS-SPME-GC-MS. Foods.

[4] Karabagias I.K., Karabagias V.K., Badeka A.V. (2021). Volatilome of White Wines as an Indicator of Authenticity and Adulteration Control Using Statistical Analysis. Aust. J. Grape Wine Res..

[5] Fonseca D., Martins N., Garcia R., Cabrita M.J. (2024). Comprehensive Two-Dimensional Gas Chromatography with a TOF MS Detector—An Effective Tool to Trace the Signature of Grape Varieties. Molecules.

[6] López R., Aznar M., Cacho J., Ferreira V. (2002). Determination of Minor and Trace Volatile Compounds in Wine by Solid-Phase Extraction and Gas Chromatography with Mass Spectrometric Detection. J. Chromatogr. A.

[7] Fernández De Simón B., Martínez J., Sanz M., Cadahía E., Esteruelas E., Muñoz A.M. (2014). Volatile Compounds and Sensorial Characterisation of Red Wine Aged in Cherry, Chestnut, False Acacia, Ash and Oak Wood Barrels. Food Chem..

[8] Šuklje K., Carlin S., Stanstrup J., Antalick G., Blackman J.W., Meeks C., Deloire A., Schmidtke L.M., Vrhovsek U. (2019). Unravelling Wine Volatile Evolution during Shiraz Grape Ripening by Untargeted HS-SPME-GC × GC-TOFMS. Food Chem..

[9] Cabrita M.J., Costa Freitas A.M., Laureano O., Borsa D., Di Stefano R. (2007). Aroma Compounds in Varietal Wines from Alentejo, Portugal. J. Food Compos. Anal..

[10] Welke J.E., Manfroi V., Zanus M., Lazzarotto M., Alcaraz Zini C. (2013). Differentiation of Wines According to Grape Variety Using Multivariate Analysis of Comprehensive Two-Dimensional Gas Chromatography with Time-of-Flight Mass Spectrometric Detection Data. Food Chem..

[11] Sagratini G., Maggi F., Caprioli G., Cristalli G., Ricciutelli M., Torregiani E., Vittori S. (2012). Comparative Study of Aroma Profile and Phenolic Content of Montepulciano Monovarietal Red Wines from the Marches and Abruzzo Regions of Italy Using HS-SPME–GC–MS and HPLC–MS. Food Chem..

[12] Petronilho S., Lopez R., Ferreira V., Coimbra M.A., Rocha S.M. (2020). Revealing the Usefulness of Aroma Networks to Explain Wine Aroma Properties: A Case Study of Portuguese Wines. Molecules.

[13] Pietra Torres M., Cabrita M.J., Gomes Da Silva M.D.R., Palma V., Costa Freitas A.M. (2011). The Impact of Malolactic Fermentation on the Volatile Composition of the Trincadeira Wine Variety: Impact of Mlf on Trincadeira Wine Volatile Composition. J. Food Biochem..

[14] Cosme F., Milheiro J., Pires J., Guerra-Gomes F.I., Filipe-Ribeiro L., Nunes F.M. (2021). Authentication of Douro DO Monovarietal Red Wines Based on Anthocyanin Profile: Comparison of Partial Least Squares—Discriminant Analysis, Decision Trees and Artificial Neural Networks. Food Control.

[15] Barreto De Oliveira J., Lemos Faria D., Fernandes Duarte D., Egipto R., Laureano O., De Castro R., Pereira G.E., Ricardo-da-Silva J.M. (2018). Effect of the Harvest Season on Phenolic Composition and Oenological Parameters of Grapes and Wines Cv. ‘Touriga Nacional’ (*Vitis vinifera* L.) Produced under Tropical Semi-Arid Climate, in the State of Pernambuco, Brazil. Ciência Téc. Vitiv..

[16] Câmara J.S., Alves M.A., Marques J.C. (2006). Changes in Volatile Composition of Madeira Wines during Their Oxidative Ageing. Anal. Chim. Acta.

[17] Rogerson F.S.S., Grande H.J., Silva M.C.M. (1999). Free and Enzyme Enhanced Monoterpenol Content of Portuguese Red Wines from the Douro Contenido en Monoterpenos Libres e Hidrolizados Por Enzimas de Vinos Tintos Portugueses del Duero Contido en Monoterpenos Libres e Hidrolizados Por Enzimas de Viños Tintos Portugueses Do Douro. Cienc. Y Tecnol. Aliment..

[18] Botelho G., Mendes-Faia A., Clímaco M.C. (2008). Differences in Odor-Active Compounds of Trincadeira Wines Obtained from Five Different Clones. J. Agric. Food Chem..

[19] Furdíková K., Machyňáková A., Drtilová T., Špánik I. (2020). Comparison of Different Categories of Slovak Tokaj Wines in Terms of Profiles of Volatile Organic Compounds. Molecules.

[20] del Barrio Galán R., Bueno-Herrera M., de la Cuesta P.L., Pérez-Magariño S. (2022). Volatile Composition of Spanish Red Wines: Effect of Origin and Aging Time. Eur. Food Res. Technol..

[21] Feher I., Magdas D.A., Dehelean A., Sârbu C. (2019). Characterization and Classification of Wines According to Geographical Origin, Vintage and Specific Variety Based on Elemental Content: A New Chemometric Approach. J. Food Sci. Technol..

[22] Minnaar P.P., Booyse M. (2016). Differentiation among Young and Market-Ready Cabernet Sauvignon, Pinotage and Shiraz Wines: Application of Canonical Discriminant Analysis Using Flavonoid and Non-Flavonoid Compositional Data. S. Afr. J. Enol. Vitic..

[23] Leder R., Petric I.V., Jusup J., Banović M. (2021). Geographical Discrimination of Croatian Wines by Stable Isotope Ratios and Multielemental Composition Analysis. Front. Nutr..

[24] Vilanova M., Genisheva Z., Masa A., Oliveira J.M. (2010). Correlation between Volatile Composition and Sensory Properties in Spanish Albariño Wines. Microchem. J..

[25] Chen K., Xue H., Shi Q., Zhang F., Ma Q., Sun J., Liu Y., Tang Y., Wang W. (2024). Geographical identification of Chinese wine based on chemometrics combined with mineral elements, volatile components and untargeted metabonomics. Food Chem. X.

[26] Slaghenaufi D., Ugliano M. (2018). Norisoprenoids, Sesquiterpenes and Terpenoids Content of Valpolicella Wines During Aging: Investigating Aroma Potential in Relationship to Evolution of Tobacco and Balsamic Aroma in Aged Wine. Front. Chem..

[27] Mendes-Pinto M.M. (2009). Carotenoid Breakdown Products the—Norisoprenoids—In Wine Aroma. Arch. Biochem. Biophys..

[28] Li Z., Howell K., Fang Z., Zhang P. (2020). Sesquiterpenes in Grapes and Wines: Occurrence, Biosynthesis, Functionality, and Influence of Winemaking Processes. Compr. Rev. Food Sci. Food Saf..

[29] Mele M.A., Kang H.-M., Lee Y.-T., Islam M.Z. (2021). Grape Terpenoids: Flavor Importance, Genetic Regulation, and Future Potential. Crit. Rev. Food Sci. Nutr..

[30] Black C.A., Parker M., Siebert T.E., Capone D.L., Francis I.L. (2015). Terpenoids and Their Role in Wine Flavour: Recent Advances: Terpenoids: Role in Wine Flavour. Aust. J. Grape Wine Res..

[31] Van Leeuwen C., Barbe J.-C., Darriet P., Destrac-Irvine A., Gowdy M., Lytra G., Marchal A., Marchand S., Plantevin M., Poitou X. (2022). Aromatic Maturity Is a Cornerstone of Terroir Expression in Red Wine: This Article Is Published in Cooperation with Terclim 2022 (XIVth International Terroir Congress and 2nd ClimWine Symposium), 3–8 July 2022, Bordeaux, France. OENO One.

[32] Ilc T., Werck-Reichhart D., Navrot N. (2016). Meta-Analysis of the Core Aroma Components of Grape and Wine Aroma. Front. Plant Sci..

[33] Garbay J., Cameleyre M., Le Menn N., Riquier L., Barbe J.-C., Lytra G. (2024). Study of the Fruity Aroma of Red Wines Made from Grape Varieties Potentially Adapted to Climate Change Using a Semi-Preparative HPLC Method. LWT.

[34] Lin J., Massonnet M., Cantu D. (2019). The Genetic Basis of Grape and Wine Aroma. Hortic. Res..

[35] Wedler H., Pemberton R., Tantillo D. (2015). Carbocations and the Complex Flavor and Bouquet of Wine: Mechanistic Aspects of Terpene Biosynthesis in Wine Grapes. Molecules.

[36] OIV Compendium of International Methods of Wine and Must Analysis. https://www.oiv.int/standards/compendium-of-international-methods-of-wine-and-must-analysis.

