# Supplementary material for: Exploring the Volatile Fingerprinting of Young Portuguese Monovarietal Red Wines by HS-SPME-GC×GC-TOFMS: A Five-Year Study
Source: Molecules. 2025 Dec 18;30(24):4814. doi: 10.3390/molecules30244814 (PMC12735745; doi:10.3390/molecules30244814)
Supplement: Supplementary file 1 [file molecules-30-04814-s001.zip › Supplementary files.pdf]

Exploring the Volatile Fingerprinting of Young Portuguese Monovarietal Red Wines by HS-SPME-GC×GC-TOFMS: A Five-Year Study

Sousa Gastão-Muchecha [1], Nuno Martins [2], Raquel Garcia [2,3] and Maria João Cabrita [2,3,*]

[1] MED (Mediterranean Institute for Agriculture, Environment and Development), IIFA (Institute for Research and Advanced Training), Universidade de Évora, Pólo da Mitra, Ap. 94, 7006-554 Évora, Portugal; sousa.gastao@uevora.pt

[2] MED (Mediterranean Institute for Agriculture, Environment and Development) & CHANGE—Global Change and Sustainability Institute, Universidade de Évora, Pólo da Mitra, Ap. 94, 7006-554 Évora, Portugal; nmartins@uevora.pt (N.M.); raquelg@uevora.pt (R.G.)

[3] Departamento de Fitotecnia, Escola de Ciências e Tecnologia, Universidade de Évora, Pólo da Mitra, Ap. 94, 7006-554 Évora, Portugal

* Correspondence: mjbc@uevora.pt; Tel.: +351-266-760-869

Supplementary Files

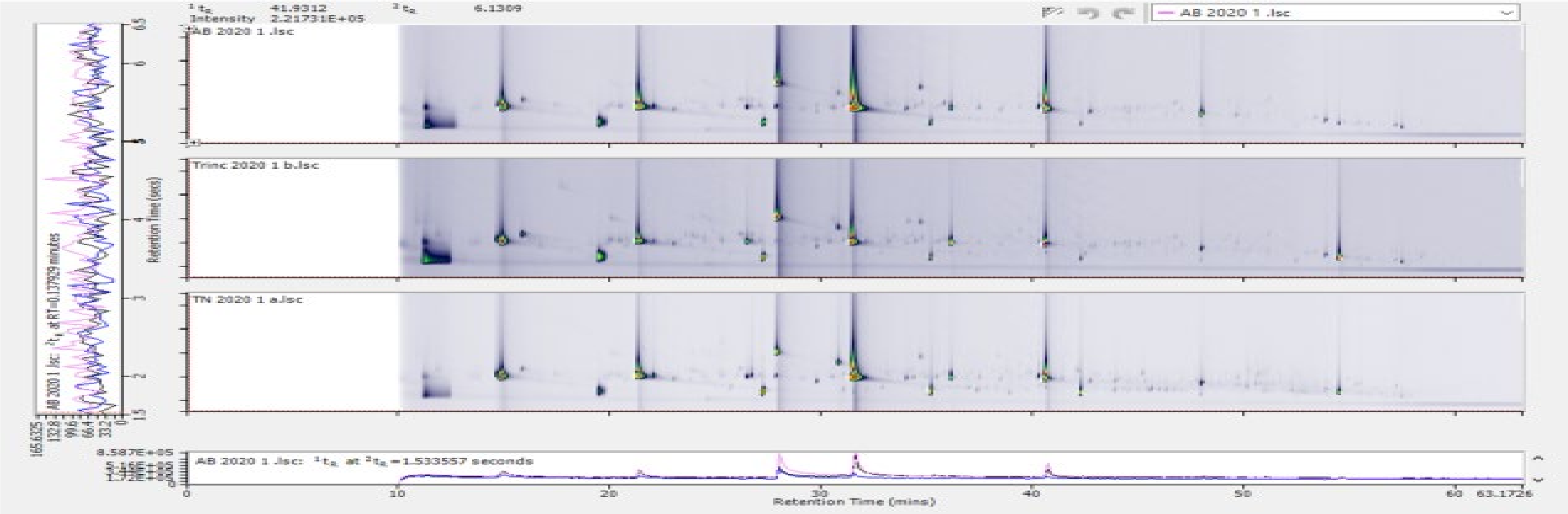

Figure S1. *Representative GC×GC-ToFMS contour plots obtained for the 2020 wines of the three grape varieties studied: Alicante Bouschet (AB), Trincadeira (Trinc), and Touriga Nacional (TN). The graphs illustrate the typical pattern of two-dimensional separation and peak distribution obtained under the analytical conditions described in Section 3.4. The lower panel shows the corresponding first-dimension chromatographic profile for one example (AB 2020).*

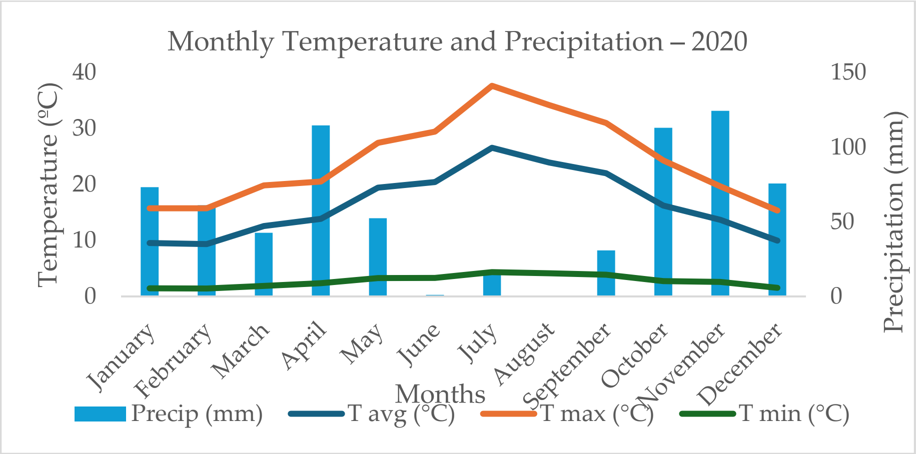

Precip = precipitation, Tavg = mean temperature average, Tmax = mean maximum temperature, Tmin = mean minimum temperature.

Figure S2. *Monthly temperature (average, maximum, and minimum) and precipitation recorded at the Mitra weather station (Évora, Portugal) during the 2020 year.*

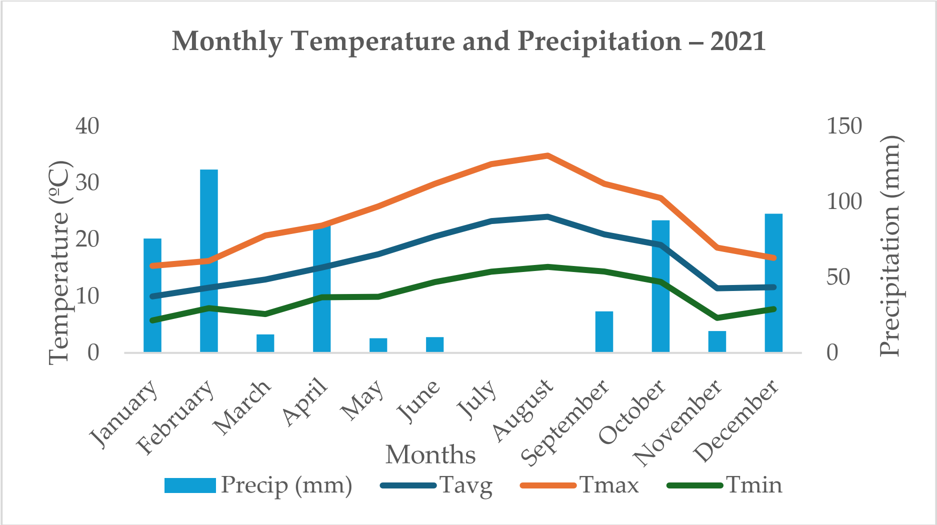

Precip = precipitation, Tavg = mean temperature average, Tmax = mean maximum temperature, Tmin = mean minimum temperature.

Figure S3. *Monthly temperature (average, maximum, and minimum) and precipitation recorded at the Mitra weather station (Évora, Portugal) during 2021.*

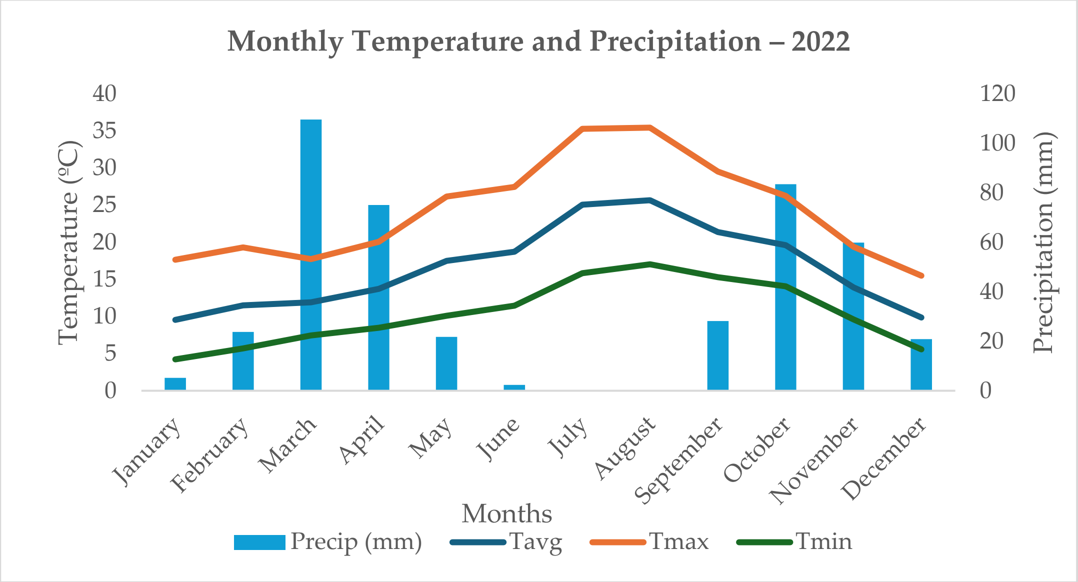

Precip = precipitation, Tavg = mean temperature average, Tmax = mean maximum temperature, Tmin = mean minimum temperature.

Figure S4. *Monthly temperature (average, maximum, and minimum) and precipitation recorded at the Mitra weather station (Évora, Portugal) during 2022.*

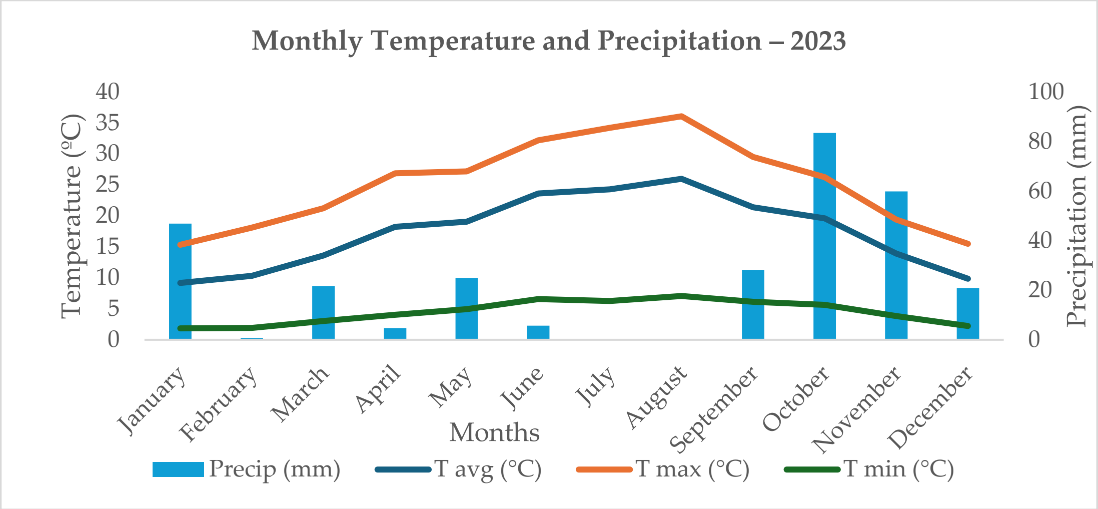

Precip = precipitation, Tavg = mean temperature average, Tmax = mean maximum temperature, Tmin = mean minimum temperature.

Figure S5. *Monthly temperature (average, maximum, and minimum) and precipitation recorded at the Mitra weather station (Évora, Portugal) during 2023.*

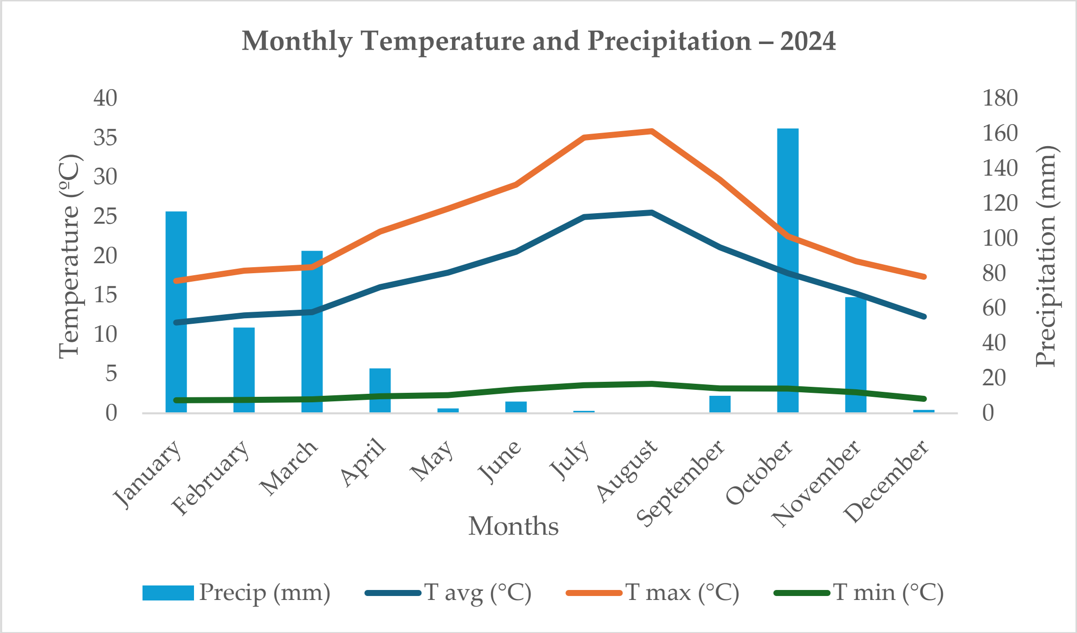

Precip = precipitation, Tavg = mean temperature average, Tmax = mean maximum temperature, Tmin = mean minimum temperature.

Figure S6. *Monthly temperature (average, maximum, and minimum) and precipitation recorded at the Mitra weather station (Évora, Portugal) during 2024.*
